# Separating Neurocognitive versus Motor Disabilities with the Digital Trail Making Test‐ Part B

**DOI:** 10.1002/alz70862_110225

**Published:** 2025-12-23

**Authors:** Ileana De Anda‐Duran, Phillip H Hwang, Elizabeth Leverant, Sheina Emrani, Louisa I. Thompson, Stacy L. Andersen, Rodney Swenson, David J. Libon

**Affiliations:** ^1^ Celia Scott Weatherhead Tulane University School of Public Health and Tropical Medicine, New Orleans, LA USA; ^2^ Department of Epidemiology, Boston University School of Public Health, Boston, MA USA; ^3^ New Jersey Institute for Successful Aging, Rowan University, Stratford, NJ USA; ^4^ University of Pennsylvania, Philadelphia, PA USA; ^5^ Alpert Medical School of Brown University, Providence, RI USA; ^6^ Boston University Chobanian & Avedisian School of Medicine, Boston, MA USA; ^7^ Department of Psychiatry, University of North Dakota Medical School, Fargo, ND USA; ^8^ Rowan‐Virtua School of Osteopathic Medicine, New Jersey Institute for Successful Aging, Stratford, NJ USA

## Abstract

**Background:**

A variety of neurocognitive/motor operations underlie successful performance on the traditional paper/pencil Trail Making Test‐Part B (TMT‐B). The current research analyzed a panel of six novel process metrics to assess how neurocognitive and motor operations on a digital version of the TMT‐B (dTMT‐B) can dissociate patients with mild cognitive impairment (MCI) versus normal cognition (NC); and relations between paper/pencil neuropsychological tests.

**Method:**

Using a digital neuropsychological battery of episodic and working memory tests, memory clinic patients (*n* = 58) were classified into groups presenting with MCI versus NC. The panel of dTMT‐B process metrics included (1) total time to completion; (2) hit duration or time spent with the pen inside target circles; (3) total number of pen strokes; (4) distance or the length of an imaginary line connecting all targets; (5) velocity or the speed pen strokes were drawn; and (6) efficiency or the straightness of pen strokes.

**Result:**

MCI patients were slightly older and less educated than NC patients; and scored lower on the MMSE. MANOVA found slower total duration (*p* <0.007; η^2^= 0.204) and hit duration (*p* <0.008; η^2^= 0.204) for MCI versus NC patients. A second MANOVA found that MCI patients produced more total pen strokes (*p* <0.044; η^2^= 0.193) and longer total distance (*p* <0.003; η^2^= 0.371) than NC patients. There were no differences for stroke velocity/efficiency. Three dTMT‐B indices measuring time (total/hit duration); pen strokes (total pen strokes/distance), and motor control (efficiency/velocity) were created. The results of a multinomial regression analysis found that only the dTMT‐B stroke index was needed to classify patients (Wald=5.09, *p* < 0.024). Partial correlations controlled for age, education, and sex found that the dTMT‐B time index was associated with tests measuring attention (WAIS‐IV Digits Forward/Trails A; r= ‐0.757, *p* <0.001); working memory (WMS‐IV Symbol Span/letter fluency); and language (r= ‐0.503, *p* <0.032). The dTMT‐B stroke index was associated with tests measuring attention (r= ‐0.648, *p* <0.009) and language (r= ‐0.582, *p* <0.023).

**Conclusion:**

These data suggest that it may be possible to fashion separate indices measuring dTMT‐B time‐based versus selected motor operations. Additional research is needed to see how well these novel dTMT‐B indices can flag emergent cognitive impairment.

